# Supplementary material for: Multi-channel polarization manipulation based on graphene for encryption communication
Source: Sci Rep. 2024 May 15;14:11155. doi: 10.1038/s41598-024-61323-9 (PMC11096339; doi:10.1038/s41598-024-61323-9)
Supplement: Supplementary file 1 — Supplementary Information. [file 41598_2024_61323_MOESM1_ESM.pdf]

Supplementary Information for

Multi-channel polarization manipulation based on graphene for encryption communication

Parsa Farzin[1], **Mohammad javad hajiahmadi**[1,*], **and Mohammad soleimani**[1]

1 School of Electrical Engineering, Iran University of Science and Technology, Tehran, 1684613114, Iran

*hajiahmadi@iust.ac.ir

The Supplementary file includes:

The detailed explanations of A) External biasing circuit; B) Floating gate and external biasing Architecture; C) Result of full structure; D) Equivalent circuit; E) Double random phase encryption (DRPE); F) The third scenario.

Supplementary Appendix A (External biasing circuit)

The complex surface conductivity of graphene is intricately linked to the concentration of free carriers, represented as "n" (cm^{-2}). The net carrier density (n) can be dynamically adjusted by applying a direct current (DC) voltage between the graphene and the silicon surface. This process enables the augmentation or reduction of charge carriers within the graphene layer as required. Following the Fowler-Nordheim tunneling mechanism [1], the graphene charge density can be represented as the integral of the tunneling current $J_{\mathrm{SiO_2}}$ within the $\mathrm{SiO_2}$,, expressed as:

$$n = \frac{1}{q}\int_0^{t_0} J_{SiO_2}\, dt \tag{s1}$$

$$J_{SiO_2} = \frac{q^3}{16\pi^2 \hbar \varphi_{SiO_2}} E_{SiO_2}^2 \exp\left(-\frac{4\sqrt{2m}\varphi_{SiO_2}^{3/2}}{3\hbar q E_{SiO_2}}\right) \tag{s2}$$

Here, $\mathrm{t_0}$ represents the duration of the voltage applied to Si, $\varphi_{\mathrm{SiO_2}} = 3.2\ eV$ [2] signifies the barrier height of $\mathrm{SiO_2}$, m denotes the effective mass of the electron, and $\mathrm{E_{SiO_2}}$ indicates the electric field intensity within $\mathrm{SiO_2}$. It is calculated by:

$$E_{SiO_2} = \frac{V_{DC}}{h_{SiO_2} + Al_2O_3\left(\varepsilon_{r,SiO_2} + \varepsilon_{r,Al_2O_3}\right)} - \frac{qn}{\varepsilon_{r,SiO_2}\left[1 + \left(\varepsilon_{r,Al_2O_3} h_{SiO_2}\right)/\varepsilon_{r,SiO_2} h_{Al_2O_3}\right]} \tag{s3}$$

Where $\mathrm{h_{SiO_2}} = 10$ nm ($\varepsilon_{\mathrm{r,SiO_2}} = 3.9$) and $\mathrm{h_{Al_2}}_{O_3} = 20$ nm ($\varepsilon_{\mathrm{r,Al_2O_3}} = 9$) are the thickness (relative permittivity) of $\mathrm{SiO_2}$ and $\mathrm{Al_2O_3}$, respectively [3], and $\mathrm{V_{DC}}$ indicates the applied voltage to the graphene layer. The connection between free carrier concentrations and chemical potential is expressed as follows [4]:

$$\mu_c = \pm\hbar v_f \sqrt{\left(\pi |n_g|\right)} \quad \text{(s4)}$$

Where $v_f = 0.9 \times 10^6\, m.s^{-1}$ is the Fermi velocity. The variation in n can be approximated by $\mathrm{n} = \varepsilon_0 \varepsilon \Delta \mathrm{V}/\mathrm{hq}$, where ε and ε0 represent the permittivities of SiO_2 and free space, respectively. Additionally, $\Delta \mathrm{V} = V_{gate} - V_{DC}$, where V_{gate} and V_{DC} correspond to the applied voltages to the gate connection and graphene surface, respectively [5]. Consequently, precise adjustment of the electrostatic voltage bias applied to graphene allows the attainment of the desired chemical potential, as indicated by the following expression:

$$\Delta V = \frac{qh\mu_c^2}{\pi\varepsilon_0\varepsilon_r\hbar^2 v_f^2} \quad \text{(s5)}$$

Supplementary Appendix B (Floating gate and external biasing Architecture)

For the purpose of modifying the charge density within each graphene layer, we utilize floating gate structures, as illustrated in Figure 2. These structures consist of Si, SiO_2, Al_2O_3, and single-layer graphene. When a positive bias voltage is applied to the upper Si layer, electrons from the lower Si layer can tunnel through the SiO_2 layer and become trapped by the graphene. This process leads to an increased charge density within the graphene layer. On the contrary, when applying a reverse bias voltage to the upper Si layer, electrons from the graphene layer can tunnel through the SiO_2 and get captured by the lower Si layer. This results in a reduction in the charge density within the graphene layer [5]. Moreover, owing to the electrical isolation of the graphene layer from the Si layers, the charge density of the graphene can remain stable over an extended period once the external bias voltage is removed. The thin layers within the floating gates play a crucial role in the design of DC bias. As the physical and dielectric properties of the metasurface remain unchanged after fabrication, reconfigurability is solely attained by modifying the bias of graphene nanoribbons. To achieve this objective, as depicted in Supplementary Figure S1, we have integrated all graphene layers into the structure using the Si-SiO_2-Graphene-Al_2O_3-Si configuration. To fine-tune the Fermi energy of graphene and thereby regulate its surface conductivity, we can leverage FPGA hardware. This hardware enables the desired electrostatic bias voltages in its output bases, facilitating the nuanced adjustment of the operational status for each layer. Because of the interconnection of each meta-atom via a gold layer, the DC voltage biasing is accomplished solely through one of the meta-atoms, effectively biasing the entire structure. This streamlined approach significantly simplifies the proposed supersurface biasing.

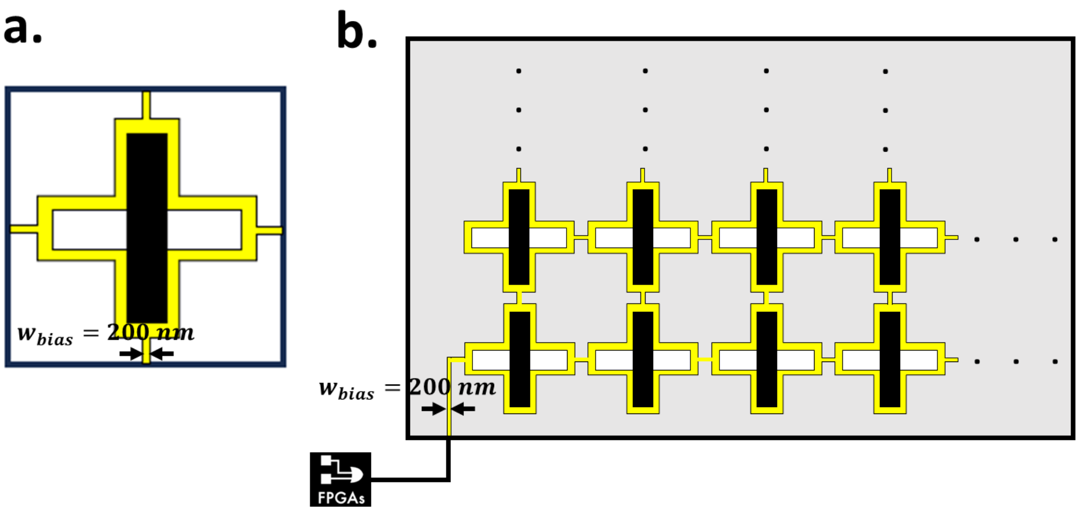

Supplementary Figure S1. A schematic representation of the proposed (a) meta-atom and (b) metasurface (Top view) is depicted, with 2D biasing grids employed to achieve amplitude modulation EM wave.

Supplementary Appendix C (Result of full structure)

When a 45°-polarized wave illuminates on the metasurface, the polarization angle of its reflected wave can be arbitrarily adjusted by controlling the amplitudes of the x- and y-polarization components. Supplementary Figure S2 illustrates the simulated results of absorption, x-, y-, and 45°-polarized reflection waves. The metasurface comprises 20×20 elements with a total size of 1000×1000 μm^2. As shown in Supplementary Figure S2(a-d), adjusting the chemical potential of Gx and Gy graphene along both x- and y-directions to the values [{0.345 eV, 0.345 eV}, {0 eV, 1 eV}, {1 eV, 0 eV}, {0 eV, 0 eV}] results in the absorption, reflected x-polarization, reflected y-polarization, and reflected 45°-polarization of the incident wave with a 45° polarization. It's important to highlight that, as depicted in Figure 2, at certain frequencies, the amplitude of the electric field (E-field) in the reflected wave exceeds 0 dB. This occurrence arises from the simulations conducted in CST software for a 45°-polarization incident on the metasurface. In these simulations, the amplitude of the E-field for both x- and y-polarizations is set to 1 V/m. Consequently, the amplitude of the incident E-field is equivalent to $\sqrt{2}$ V/m or 3.01 V/m in dB, as show in Supplementary Figure S3. To measure the amplitude of the reflected electric field, we utilized a probe in the farfield of the CST software program.

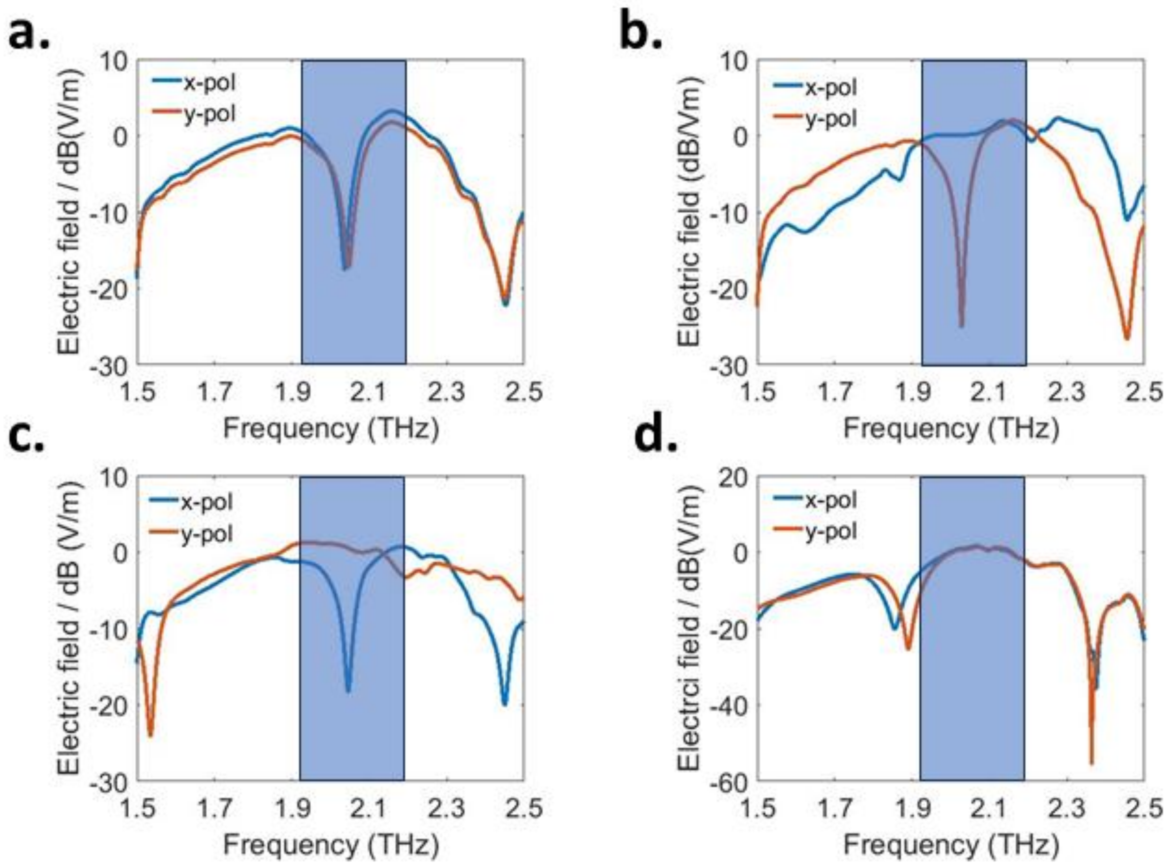

Supplementary Figure S2. The simulated amplitude of the E-field reflected for four amplitude modulation states: a) absorption, b) x-polarization, c) y-polarization, and d) 45°-polarization.

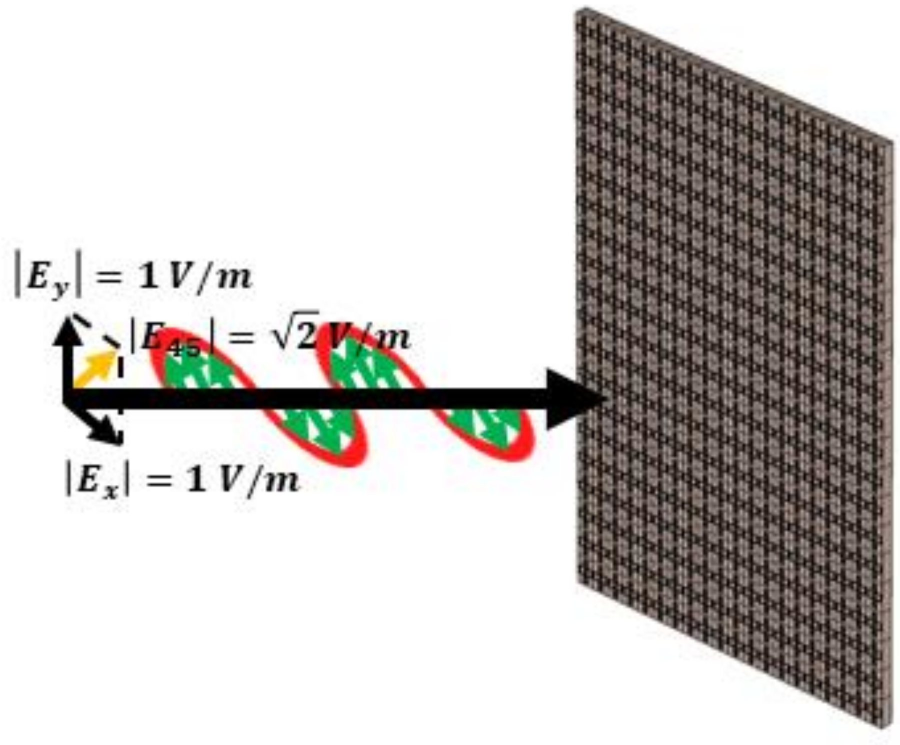

Supplementary Figure S3. The amplitude of the incident E-field on the metasurface.

Supplementary Appendix D (equivalent circuit)

In the suggested equivalent circuit as per [6], we have represented the graphene-gold combination as an integrated R-L-C, denoted with the label "GG". Owing to the unique arrangement of graphenes in both horizontal and vertical directions, the radiation waves with x- and y-polarizations exert distinct influences on the reflected wave. Hence, we have employed two suggested equivalent circuits. When a wave with y-polarization illuminates the metasurface, the graphene nanoribbon of the first layer, extending horizontally, does not influence the incident wave. Instead, only the resistive effect of gold affects the wave. Consequently, in the suggested equivalent circuit, this layer is treated as an open circuit, as we have considered. The same approach applies to the x-polarized wave.

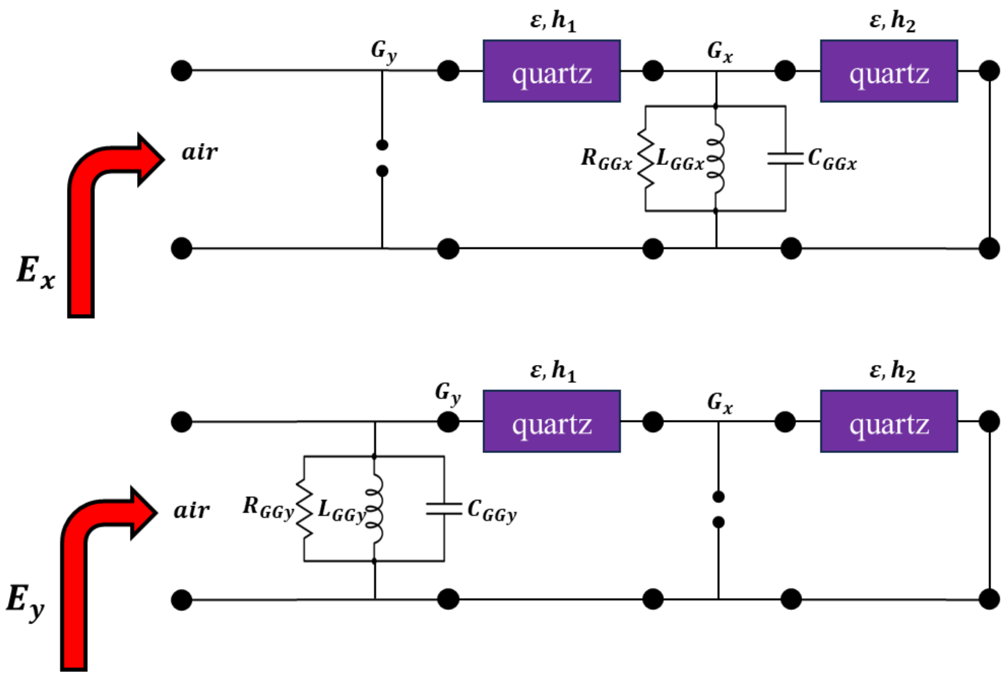

Supplementary Figure S4. The proposed circuit model for prediction of the reflection spectra of the employed meta-atoms.

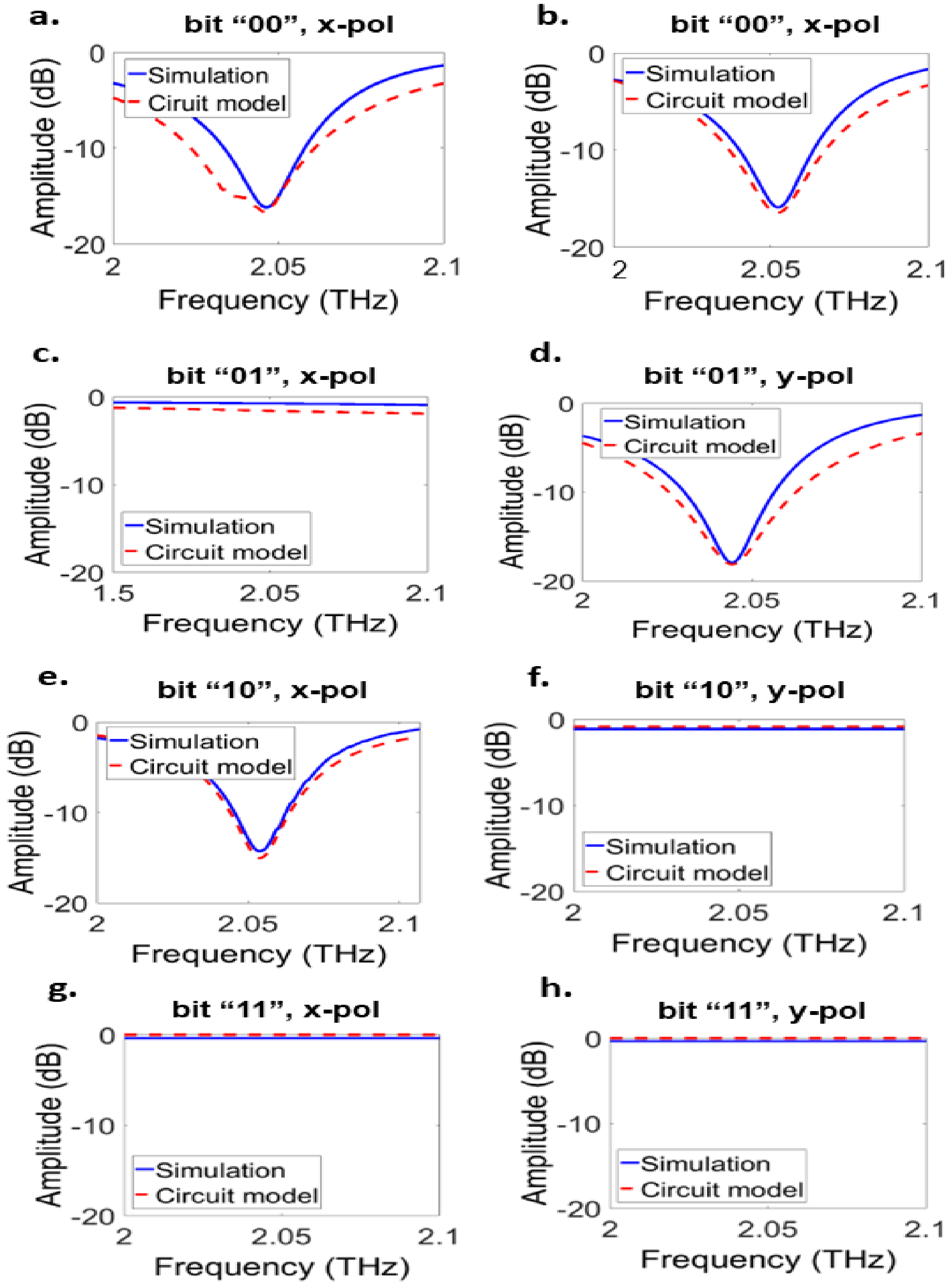

Supplementary Figure S5. Comparison between full-wave simulations and the proposed circuit model. Bit "00": (a and b) absorption mode in both x- and y-modes respectively. Bit "01": (c) reflection mode for x-polarization and (d) absorption mode for y-polarization. Bit "10": (e) absorption mode for x-polarization and (f) reflection mode for y-polarization. Bit 11: (g and h) reflection mode for both x- and y-polarizations, respectively.

Supplementary Table S1. The values of the circuit components for the employed graphene metasurfaces.

Layer	state	$R_{GG}(\Omega)$	L_{GG} (pH)	C_{GG} (pf)
First layer (x-pol)	Absorption	37	84	72
	Reflect x-pol	47	84	85
	Reflect y-pol	6	84	64.6
	Reflect 45-pol	35	84	72.3
second layer (y-pol)	Absorption	38	108	61.3
	Reflect x-pol	40	105	70.1
	Reflect y-pol	39	103	59.3
	Reflect 45-pol	7	105	55.1

Supplementary Appendix E (Double random phase encryption (DRPE))

Optical cryptographic techniques found extensive application in image security, given their capacity to efficiently handle large volumes of data through image processing algorithms. Image encryption, in particular, has garnered significant attention, especially since the introduction of double phase random encryption (DRPE) in [7] which to be a distinctive approach for optically encoding an image [8]. As show in Supplementary Figure S4, to encrypt the transmitted information using the DRPE algorithm, the initial input image "Richard Feynman" is encrypted by employing two statistically independent random phase keys and undergoing two Fourier transforms to introduce constant white noise. DRPE utilizes techniques such as the 4f system and Fourier Transform (FT), allowing for the encryption of both spatial and spectral information [7]. In DRPE, a plaintext image undergoes random phase modulation through two random phase masks—RPM1 in the spatial plane and RPM2 in the Fourier plane. During the encryption process, a plaintext image, $f(x,y)$, is multiplied by the initial random phase mask, $exp\{jR_1(x,y)\}$, in the spatial plane as follows [8]:

$$f_m(x,y) = f(x,y)\,.exp\{jR_1(x,y)\} \quad \text{(S1)}$$

where x and y denote the spatial coordinates. After the complex amplitude $f_m(x, y)$ is subjected to a Fourier transform, the resulting transformed image $F_m(\mu, \nu)$ is multiplied by the second random phase mask (RPM2), denoted as $exp\{jR_2(x, y)\}$, used as the encryption key. The complex amplitude image then undergoes an inverse Fourier transform to obtain the ciphertext image, $c(x, y)$, at the spatial plane as follows [8]:

$$c(x, y) = IF[F_m(\mu, \nu). exp\{jR_2(x, y)\}] \tag{S2}$$

Here, IF[*] represents the operator for inverse Fourier transform, while μ and ν denote the spatial frequency coordinates. Decryption is achieved through the inverse process and the application of the corresponding key [9].

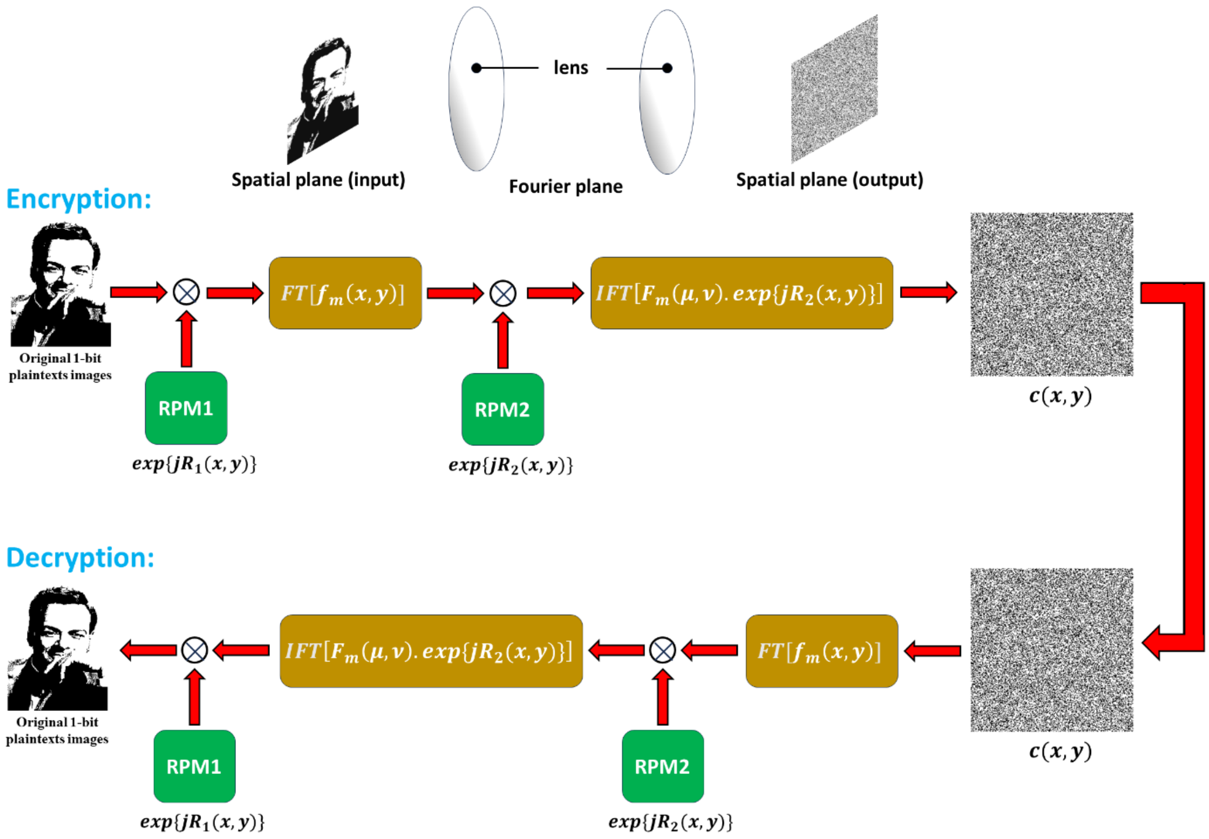

Supplementary Figure S6. Double random phase encryption and decryption.

Supplementary Appendix F (The third scenario)

We investigated the scenarios involving the transmission of one and two photos. Subsequently, we will explore the scenario of transmitting more than two images. Initially, we employ the DRPE algorithm to encrypt all three original images (cat, mandrill, and Einstein). Consequently, three 1-bit ciphertext images are generated. Similar to scenario 2, the conversion of each of the three ciphertext images into a sequence of binary symbols, denoted by "0" and "1," takes place. These symbol sequences are transmitted through the proposed metasurface, utilizing three polarization channels (x-, y-, and 45°-polarization) with varying amplitudes within a time duration of “T” seconds.

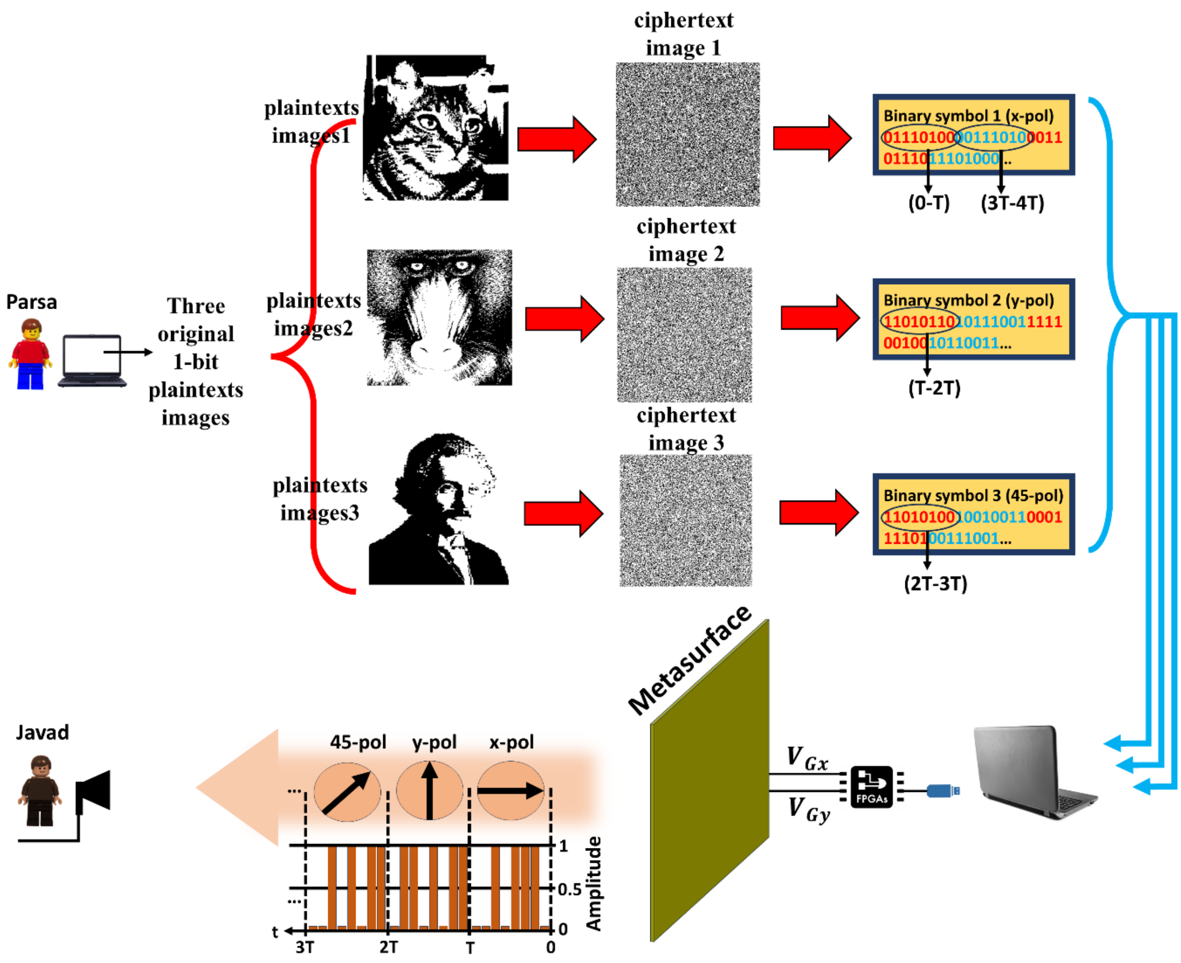

Supplementary Figure S7. Encrypting process of the third scenario: Encrypting three pictures at the same time

References

[1] Lenzlinger, M., and E. H. Snow. "Fowler- Nordheim tunneling into thermally grown SiO2." *Journal of Applied physics* 40.1 (1969): 278-283.

[2] Peng, Xi-Liang, et al. "An active absorber based on nonvolatile floating-gate graphene structure." *IEEE Transactions on Nanotechnology* 16.2 (2017): 189-195.

[3] Li, Yan, et al. "Graphene-based floating-gate nonvolatile optical switch." *IEEE Photonics Technology Letters* 28.3 (2015): 284-287.

[4] Huang, Yuanyuan, et al. "Tunable circular polarization conversion and asymmetric transmission of planar chiral graphene-metamaterial in terahertz region." *Carbon* 119 (2017): 305-313.

[5] Peng, Xi-Liang, et al. "An active absorber based on nonvolatile floating-gate graphene structure." *IEEE Transactions on Nanotechnology* 16.2 (2017): 189-195.

[6] Pruneanu, Stela, et al. "Novel graphene-gold nanoparticle modified electrodes for the high sensitivity electrochemical spectroscopy detection and analysis of carbamazepine." *The Journal of Physical Chemistry C* 115.47 (2011): 23387-23394.

[7] Refregier, Philippe, and Bahram Javidi. "Optical image encryption based on input plane and Fourier plane random encoding." *Optics letters* 20.7 (1995): 767-769.

[8] Liu, Zhe, Mee Loong Yang, and Wei Qi Yan. "Image encryption based on double random phase encoding." *2017 International Conference on Image and Vision Computing New Zealand (IVCNZ)*. IEEE, 2017.

[9] Nakano, Kazuya, and Hiroyuki Suzuki. "Analysis of singular phase based on double random phase encoding using phase retrieval algorithm." *Optics and Lasers in Engineering* 134 (2020): 106300.
